# Coronary endothelial function using 3T MRI is inversely related to body mass index

**DOI:** 10.1186/1532-429X-14-S1-P73

**Published:** 2012-02-01

**Authors:** Allison Hays, Sahar Soleimanifard, Michael Schär, Gary Gerstenblith, Kerry Stewart, Matthias Stuber, Robert G Weiss

**Affiliations:** 1 Medicine, Johns Hopkins, Baltimore, MD, USA; 2 Radiology, Johns Hopkins, Baltimore, MD, USA; 3 Philips Healthcare, Cleveland, OH, USA; 4 Radiology, Centre Hospitalier Universitaire Vaudois, Lausanne, Switzerland

## Summary

Using 3T MRI combined with isometric handgrip exercise to quantify coronary endothelial-dependent vasoreactivity, we observed significantly reduced coronary EndoFx in overweight/obese individuals compared to normal weight adults and detected a significant inverse relationship between BMI and % CSA, a measure of endothelial independent vasoreacivity.

## Background

Although obesity is a known risk factor for future cardiovascular events [1], its relationship to coronary endothelial function, which is impaired in early atherosclerosis, is not completely understood. By means of previously described non-invasive 3T MRI methods combined with isometric handgrip to assess endothelial-dependent coronary vasoreactivity [2], we sought to test the hypothesis that coronary endothelial function (EndoFx) is reduced in obese, non-diabetic individuals when compared to healthy non-obese volunteers and that body mass index (BMI) is inversely related to EndoFx.

## Methods

14 overweight and obese non-diabetic adults defined as BMI >25 (BMI=32.3±4.4, mean±SD, 9 women) and 10 healthy non-overweight adults (BMI=22.9±2.8, 7 women) with no history of cardiovascular disease or risk factors were imaged using a 3T MRI scanner (Achieva, Philips, Best, NL) and a 32-element cardiac coil for signal reception. To measure endoFx, spiral coronary MRI was performed before and during isometric handgrip exercise, an endothelial-dependent stressor, and coronary cross-sectional area (CSA) and coronary blood velocity (CV) changes were measured and coronary blood flow (CBF) calculated as previously reported [2].

## Results

**Figure 1:**
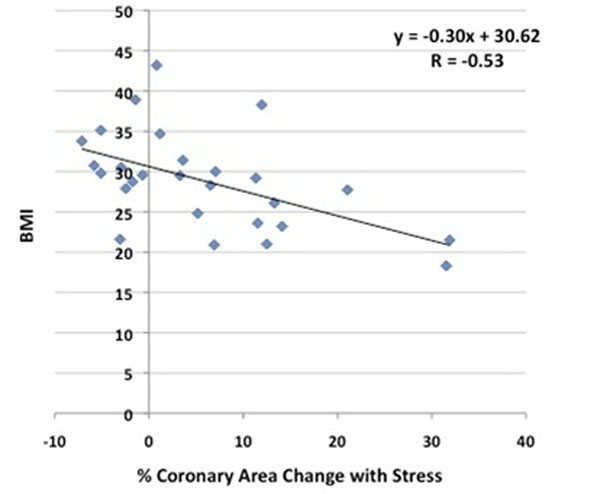
% coronary artery area change with isometric handgrip stress is inversely related to body mass index (BMI) in adults, N=27, p=0.004.

17 coronary arteries in the overweight patients and 10 coronaries in the normal weight group had adequate image quality for coronary EndoFx measurements. There was no significant difference in mean age between the healthy (50±10.2 years) and overweight/obese group (53±9.9 years, p=0.83). The stress-induced change in CSA was significantly higher in healthy non-obese adults (12.1±11.9%) than in the obese individuals (2.2±7.7%, p=0.02). Although there was a trend toward lower CBF change with stress in the obese group (17.4±32.5%) compared to the healthy group (30.0±27.1%, p=0.21), it was not statistically significant, and there was no difference in % CV change between the two groups (healthy: 14.7±13.2%, obese: 13.3±24.8, p=0.69). In the entire population, we detected a significant inverse relationship between BMI and % CSA change with stress (r=-0.53, p=0.004, see Fig 1).

## Conclusions

Using 3T MRI combined with isometric handgrip exercise to quantify coronary endothelial-dependent vasoreactivity, we observed significantly reduced coronary EndoFx in overweight/obese individuals compared to normal weight adults and detected a significant inverse relationship between BMI and % CSA. The present findings demonstrate that it is possible to non-invasively evaluate coronary EndoFx in this population and that obesity is significantly related to coronary endothelial dysfunction. This noninvasive MR approach permits the serial study of EndoFx in obese patients and may offer important insights into risk stratification or the effects of therapeutic or lifestyle interventions on obesity.

## Funding

NIH/NHLBI (ROI-HL084186), Reynolds Foundation and American Heart Association.
